# *Mycobacterium marinum* hand infection: a case report and literature review

**DOI:** 10.3389/fmed.2024.1433153

**Published:** 2024-08-09

**Authors:** Chunping Liu, Jiahao Hao, Minghui Song, Jiaqing Ye, Cuiying Zheng, Yinqi Huang, Zhongjun Feng, Ruiping Jiang, Yan Shi, Weili Gao, Huifen Zuo, Zhenjun Zhao, Lijie Zhang

**Affiliations:** ^1^Department of Dermatology, Hebei Medical University Third Hospital, Shijiazhuang, China; ^2^Department of Clinical Laboratory, Hebei Medical University Third Hospital, Shijiazhuang, China; ^3^Hebei Key Laboratory of Intractable Pathogens, Shijiazhuang Center for Disease Control and Prevention, Shijiazhuang, China; ^4^Department of Clinical Laboratory, Hebei Yiling Hospital, Shijiazhuang, Hebei, China

**Keywords:** *Mycobacterium marinum*, skin infection, acid-fast stain, nontuberculous mycobacteria, opportunistic pathogen

## Abstract

*Mycobacterium marinum*, a photochromogenic, slow-growing mycobacterium, thrives in both marine and freshwater environments. Optimal growth occurs between 25°C and 35°C, with survival becoming challenging above 37°C. Typically, *M. marinum* enters the body via skin abrasions, often leading to infections of the upper extremities. Diagnosis of *M. marinum* infection is frequently challenging and delayed due to the difficult pathogen identification. At present, a standardized treatment protocol has yet to be established. Presented herein is a case study detailing an infection of the right hand's middle finger caused by *M*. marinum. Notably, his occupation as a chef, handling fish and seafood post-injury, was a significant factor. Histological examination of the skin biopsy and positive acid-fast staining were consistent with a diagnosis of mycobacterial infection. Pathological examination confirmed a skin infection with infectious granuloma, and tissue section acid-fast staining revealed acid-fast bacill. Cultures on Columbia blood agar yielded rough, flattened, yellow-fleshy colonies after 10 days, which was identified as *M. marinum* through 16S rRNA sequencing. The patient responded well to a 3-month regimen of oral moxifloxacin (0.4 qd) and linezolid (0.6 qd), resulting in rash resolution and pain relief, with no recurrence observed for 1-year follow-up. This report presents the first documented acid-fast staining images of *M. marinum* tissue sections and colony morphology photographs, offering an in-depth view of *M*. marinum's morphological characteristics. It aims to enhance awareness of *M. marinum* infections, underscore the necessity for clinicians to delve into patient histories, and provide a review of the clinical manifestations, diagnostic techniques, therapeutic approaches, and pathogenic mechanisms associated with *M*. marinum.

## 1 Introduction

*Non-tuberculous mycobacteria* (*NTM*) are a group of mycobacteria widely distributed in the natural environment and have been isolated from air, soil, dust, plants, natural and drinking water sources, including biofilms, wild animals, milk, and food products (1–4). In recent years, the incidence and prevalence of *NTM* infections have been on the rise due to many factors including an aging population, increased numbers of individuals with compromised immune systems, abuse of antibiotic, and advancements in molecular biology identification techniques (4). Skin infections caused by *NTM* are increasingly recognized and pose a significant public health challenge. *M*.marinum first isolated from fish in 1926 by J.D. Aronson, was later identified as a human pathogen by Linell and Norden in 1951 (5, 6). According to Runyon's classification, it belongs to photochromogenic Group I *NTM*, with an optimal growth temperature around 30°C (7). *M*.marinum, also known as atypical mycobacterium or environmental isolate, is commonly found in disinfected saline and seawater reservoirs worldwide, leading to swimming pool or aquarium granuloma (8). Infections most commonly occur through skin wounds or abrasions. The skin lesions associated with *M*. marinum infection are typically solitary or multiple red papules, nodules, or ulcers, commonly found on the elbows, knees, hands, and feet. The diagnosis of *M*. marinum infection requires clinical foresight, as it is a slow-growing mycobacterium that requires extended culture times for positive results. Diagnosis is based on a combination of patient history, clinical presentation, and laboratory tests, including bacterial culture, molecular biology, and histopathology. There is no standardized treatment protocol for *M*. marinum infection; therapeutic strategies must be individualized based on infection location, severity, and patient immune status. This case report presents a chef with no notable medical or immunosuppressive history, who developed a skin infection by *M*. marinum following an outdoor injury. The patient achieved successful treatment outcomes with oral moxifloxacin and linezolid, showing no signs of recurrence during follow-up. This article outlines a practical clinical treatment approach and delves into the patient's clinical symptoms, diagnostic tests, outcomes, and pertinent literature, contributing to the broader understanding and management of *M*. marinum infections.

## 2 Case presentation

### 2.1 Patient characteristics

**Figure 1:**
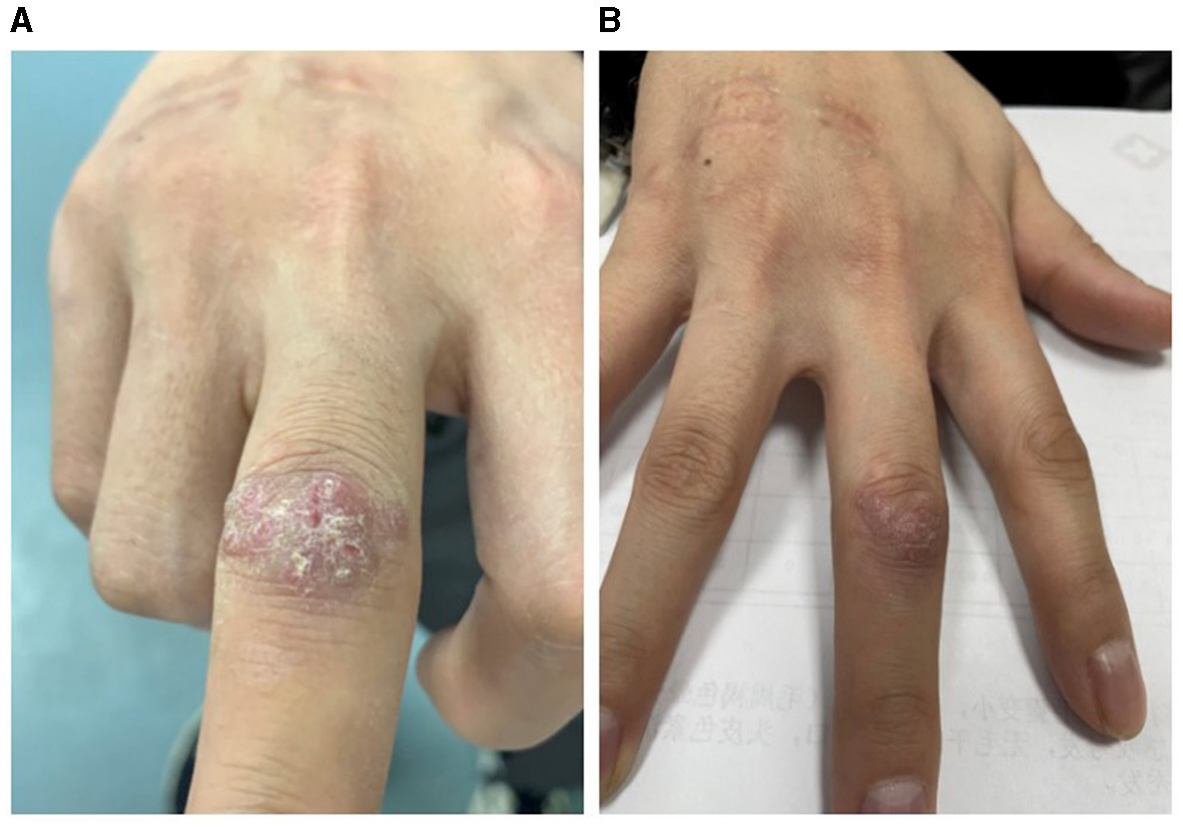
Photos of the patient's right hand. **(A)** Pre-treatment, the patient's right middle finger joint showed an irregular plaque with scales. **(B)** Post-treatment, the patient's right middle finger joint rash had mostly flattened.

A 22-year-old male patient presented with a history of a finger injury sustained in October 2022 during an outdoor excursion in a wooded area. In the aftermath, he engaged in processing fish and seafood. Subsequently, the patient developed localized papules and nodules with associated pain. The condition progressed to a more severe rash with local purulent discharge. Despite treatment with an unspecified topical medication, there was no improvement. He sought medical attention at Hebei Medical University Third Hospital on January 3, 2023. Dermatological examination revealed an irregular plaque with scales on the extensor side of the right middle finger joint (see Figure 1A). Local tissue was collected for pathological examination and microbial culture, with the clinical suspicion of *Sporothrix schenckii* infection prompting extended culture duration.

### 2.2 Laboratory examination

**Figure 2:**
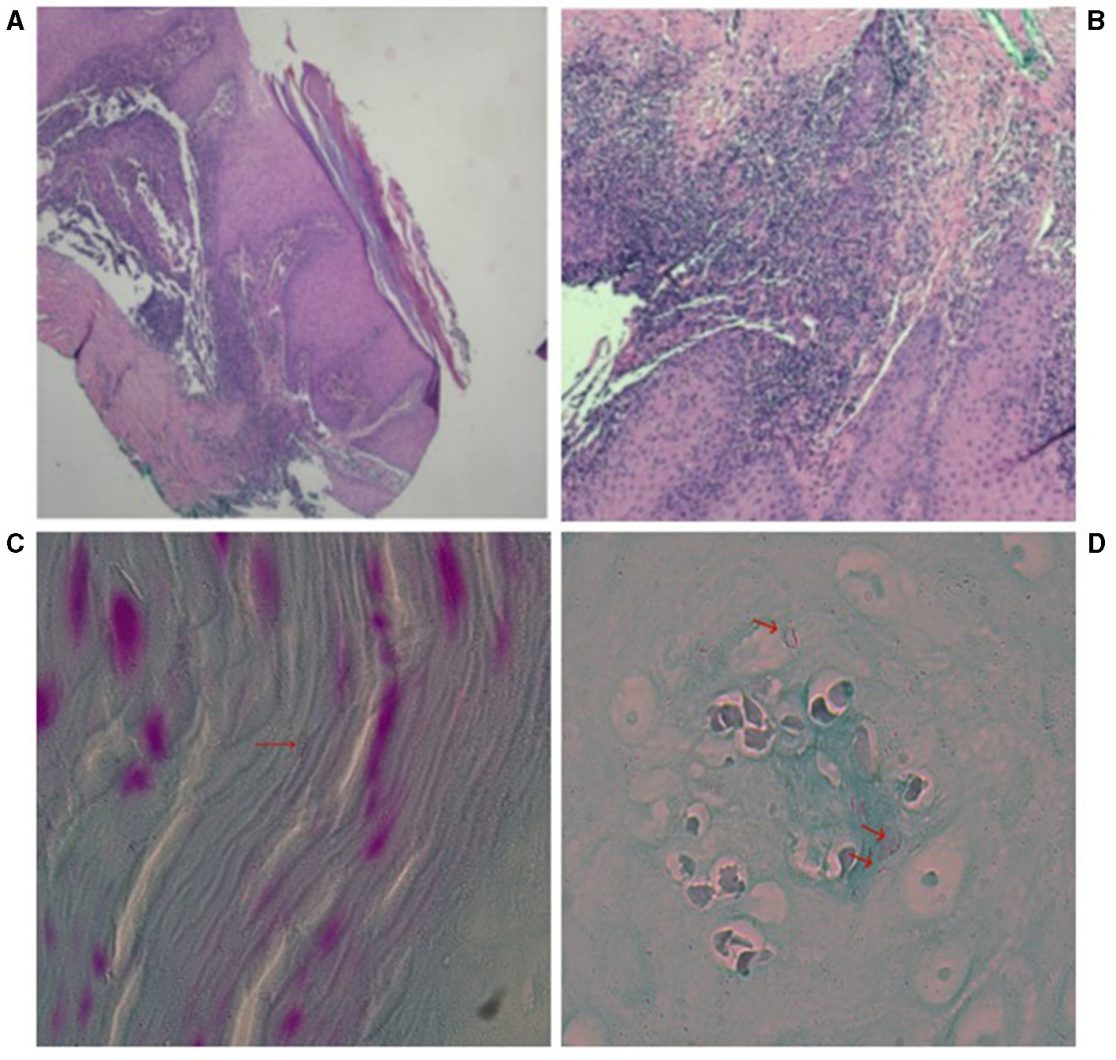
The pathology photograph of the dorsal aspect of the right middle finger joint. **(A)** Hyperplasia of the squamous epithelium of the epidermis with hyperkeratosis. H&E, magnification, 40X **(B)** superficial dermal acute inflammation with localized abscess formation and extensive lymphocytic infiltration. H&E, magnification, 100X **(C, D)** high magnification of acid-fast stained pathological tissue sections from the right middle finger joint showing curved red bacilli.

**Figure 3:**
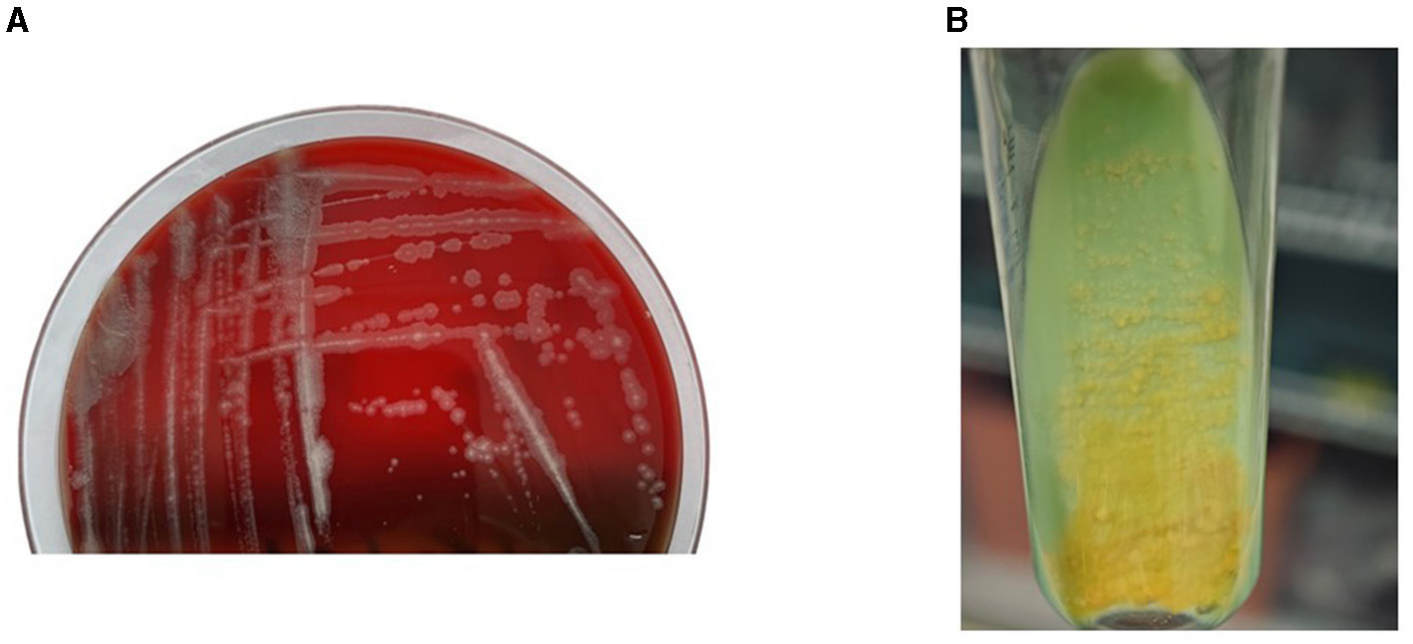
**(A)** Rough colonies grown on Columbia blood agar. **(B)** Colonies were subcultured on Lowenstein-Jensen solid medium and incubated at 28°C for 7 days, resulting in the development of yellow colonies under light exposure.

The histopathological findings from January 5, 2023, are as follows: The histopathology of the right middle finger joint's extensor side revealed hyperplastic squamous epithelium with hyperkeratosis. The superficial dermis showed acute inflammation with localized abscess formation and extensive lymphocytic infiltration (see Figures 2A, B). Microbial examination: At the time of medical consultation, pus was collected from the patient's right middle finger for general bacterial smear and culture. Acid-fast staining of pathological tissue sections revealed acid-fast bacilli (see Figures 2C, D). After 10 days of incubation, colonies emerged. Following a week of sub-culturing on Columbia blood aga rough, flattened, yellow-fleshy colonies were observed (see Figure 3A). Colonies were sub-cultured on Lowenstein-Jensen solid medium and incubated at 28°C for 7 days, resulting in the development of yellow colonies under light exposure (see Figure 3B). The culture smear showed Gram-positive rods, acid-fast staining positive. Based on the culture characteristics and staining, it is highly suspected to be an *NTM*. The laboratory also identified the isolated bacteria as *M*. marinum through 16S gene sequence analysis. These photos are of significant importance in the diagnostic process. The histopathological acid-fast staining images and colony morphology photos provide a detailed depiction of the characteristics of *M. marinum* infection for the first time, aiding clinicians in better identifying and diagnosing such infections. Additionally, these photos offer valuable reference material for the academic community in clinical microbiology, showcasing specific details of laboratory diagnosis and differential diagnosis. The nucleotide homology reached 99.86% with *M*. marinum from the NCBI database (MK411581.1). We submit the sequences of *M*. marinum to the GenBank database in NCBI with the accession number of OR876336 (https://www.ncbi.nlm.nih.gov/nuccore/OR876336).

### 2.3 Treatment

While awaiting laboratory results, the patient received standard postoperative care without any special treatment. Two weeks post-suture removal, spontaneous improvement in the local lesion was noted. Considering the patient's young age, good health, and absence of immunodeficiency, coupled with the infection's location on the extensor side between the second and third interphalangeal joints of the right middle finger, surgical excision was deemed to have a significant aesthetic and functional impact on finger flexion. As the rash showed signs of subsiding, the patient was administered an oral regimen of linezolid (0.6 g daily) and moxifloxacin (0.4 g daily). After 3 months, the rash flattened, and the pain resolved. With an additional 2 weeks of treatment, the rash substantially receded (see Figure 1B), with no recurrence observed for 1-year follow-up.

## 3 Conclusion

*M. marinum* infections, while not exceedingly common, are also not rare. Since the initial human skin infection case linked to *M. marinum* in a contaminated swimming pool reported by Linell and Norden in Sweden in 1951, many case reports and series have been published (9–17). The most common route of infection is through injuries caused by contaminated water or marinum products, especially prevalent in aquarium environments. Here are some possible occupations at risk: (1) Aquarium staff or enthusiasts (16, 18–22). (2) Fishermen and those handling fish and other seafood (including chefs, fishmongers, etc.) (7, 18, 23, 24). (3) Individuals engaged in water activities (10, 20, 25, 26). Reports indicate that fish pedicures can also lead to infection (27). Due to its growth characteristics, *M*. marinum is most active in water temperatures of 28–32°C and struggles to survive above 37°C. Therefore, it primarily infects the skin and fascia of the hands or lower limbs, especially when abrasions or wounds are present, with lesions appearing after a prolonged incubation period. The infection typically presents with red papules, nodules, ulcers, lymphangitis, cellulitis, or lymphadenopathy, particularly in the upper limbs. Extracutaneous infections may include tenosynovitis, bursitis, arthritis, and osteomyelitis. Individuals with compromised immune systems, such as HIV patients or organ transplant recipients, may experience more severe systemic infections (28). Treatment for *M*. marinum infection includes antibiotics and surgery, tailored to the patient's specific situation and the severity of the infection. Skin and soft tissue infections are generally treated with oral or intravenous antibiotics, with common antibiotics including macrolides (such as clarithromycin or azithromycin), tetracyclines (such as doxycycline or minocycline), compound sulfonamides, quinolones (such as ciprofloxacin or levofloxacin), aminoglycosides (such as amikacin or gentamicin), rifampicin, and ethambutol. Linezolid, an oxazolidinone antibiotic, has been reported to exhibit *in vitro* antibacterial activity against *M*.marinum at clinically achievable concentrations, but prolonged use may result in side effects such as numbness (29–32). Typically, a combination of two or more antibiotics is required, with treatment lasting at least 3 months until complete healing of the skin lesion. In certain cases, such as hand infections, surgical procedures may be necessary to remove infected tissue. For patients with compromised immune systems, treatment may need to be prolonged, and sometimes continuous prophylactic treatment is necessary to prevent recurrence of the infection (33). Therefore, early diagnosis and treatment of *M*. marinum infection are crucial to avoid serious complications and sequelae. *M. marinum* infection can be confused with other conditions due to similar clinical presentations. Differential diagnoses include the following aspects: (1) other *NTM* such as *M. chelonae, M. ulcerans, M. haemophilum*, and *M. fortuitum*. (2) Sporotrichosis caused by *Sporothrix schenckii*. (3) Non-infectious diseases such as contact dermatitis, eczema, pompholyx, psoriasis, sarcoidosis, skin tumors, and foreign-body reactions. Diagnostic methods are shown in Table 1. Accurate diagnosis of *M. marinum* requires multiple methods: (1) Histopathology: Granulomatous inflammation is usually observed. (2) Microbiological Culture: Cultures are typically positive for *M. marinum*. (3) 16S rRNA Gene Sequencing and MALDI-TOF Mass Spectrometry: These molecular techniques are crucial for identifying specific mycobacteria. (4) Acid-Fast Staining: Used to detect mycobacteria in tissue samples. Challenges in treating *M.marinum* infections include: (1) Diagnostic Delays: The average time to diagnosis can be long. (2) Diagnostic Complexity: The clinical manifestations of *M.marinum* infection are diverse. Eczematous changes may occur, and there may be only scaling and crusting without obvious inflammatory components and infiltration. (3) Treatment Resistance: Some strains may exhibit resistance to common antibiotics, necessitating tailored treatment regimens. Post-treatment sequelae may include: (1) Persistent Skin Lesions: Some patients may experience residual skin changes even after successful treatment. (2) Functional Impairment: Particularly in severe cases with delayed diagnosis and treatment (34). Therefore, Diagnosis requires clinical foresight and a complete patient history, combined with biopsy and subsequent microbiological confirmation. Jernigan et al. reported that the incubation period for *M*. marinum infection averages 21 days, aiding clinicians in patient history inquiry (35). In recent years, the pathogenic mechanism of *M*. marinum has been a hot topic, mainly because it is the closest genetic relative to the *Mycobacterium tuberculosis complex*. Both have a considerable degree of common genetic procedure and host immune response, but *M*. marinum is easier to manipulate on the experimental bench (36, 37). Mycobacteria utilize five independent Type VII secretion systems, named ESX-1 to ESX-5, which are essential for specific functions (38). Two of these systems, ESX-3 and ESX-5, are indispensable for the bacterium's extracellular growth, making them interesting targets for new antibiotic development (39). Researchers studying *M*. marinum infection in zebrafish models have better proposed insights into the pathogenic mechanisms of the Mycobacterium tuberculosis complex. Studies have shown that the ESX-1 system plays a significant role in the pathogenicity of *M*. marinum. This system is a specialized secretion system that is crucial for the virulence of *M*. marinum, and Ho et al. discovered the dysregulation of the ESX-5 secretion by novel 1,2,4-triazoles against *M. marinum* (40). By studying this system, we can gain a deeper understanding of the pathogenic mechanisms of *M*. marinum and the *Mycobacterium tuberculosis complex*, providing important guidance and insights for the development of new treatment methods and prevention strategies.

**Table 1:** Differential diagnosis for *M. marinum* infection.

**Disease type**	**Specific disease**	**Diagnostic methods**
*NTM* infections	*M. chelonae, M. ulcerans, M. haemophilum, M. fortuitum*	**Microbiological Culture:** Using selective media containing antibiotics, with a longer culture time **Molecular Diagnostics:** Rapid identification of *NTM* species **Histopathology:** Detection of mycobacteria through biopsy and acid-fast staining
Sporotrichosis	Sporothrix schenckii	**Microbiological Culture**: Culturing fungi from clinical specimens **PCR and Molecular Diagnostics**: Determining the specific type of infection **Immunological Tests**: Enzyme-linked immunosorbent assay (ELISA) or latex agglutination test **Histopathology**: Detection of fungi through biopsy and immunohistochemistry
Non-infectious diseases	Contact dermatitis	**History and Physical Examination**: Assessing exposure to new chemicals, cosmetics, skincare products, or other potential allergens **Patch Test**: Applying common allergens to the skin and observing for local allergic reactions
	Eczema	**History and Physical Examination**: Inquiring about family history and personal allergy history, such as asthma or allergic rhinitis **Skin Prick Test**: Testing for reactions to specific allergens **Serum IgE Testing**: Elevated serum IgE levels are common in eczema patients
	Pompholyx	**History and Physical Examination**: Assessing for a history of recurrent small blisters **Skin Biopsy**: Observing skin tissue under a microscope, with characteristics of intraepidermal and subepidermal vesicles
	Psoriasis	**History and Physical Examination:** Inquiring about family history, as psoriasis has a genetic predisposition **Skin Biopsy:** Observing for hyperkeratosis, subcorneal microabscesses, and elongated dermal papillae under a microscope
	Sarcoidosis	**History and Physical Examination:** Evaluating clinical symptoms and signs **Imaging Studies:** Chest X-ray and high-resolution CT scan **Histopathology:** Detecting non-caseating granulomas through biopsy **Laboratory Tests:** Elevated serum ACE levels
	Skin tumors	**History and Physical Examination:** Assessing the morphology and distribution of skin lesions **Skin Biopsy**: Determining the type of tumor through histopathological examination **Imaging Studies:** CT or MRI scans if metastatic tumors are suspected
	Foreign-body reactions	**History and Physical Examination:** Assessing for a history of trauma or surgery **Imaging Studies:** X-ray or ultrasound to detect foreign bodies **Histopathology:** Observing the inflammatory response around the foreign body through biopsy

This case suggests:

1. Individuals with normal immune function can contract opportunistic *NTM* infections following trauma. Increased awareness and preventive measures should be emphasized for those frequently in contact with seawater, freshwater environments and seafood, such as fishermen, aquarium workers, water sports enthusiasts and chef. Moreover, with the rise in marinum activities and the consumption of seafood, public awareness and prevention of *M*. marinum infections should also be strengthened.
2. The clinical foresight of physicians regarding special bacterial infections (as in this case where the initial suspicion was a filamentous fungal infection, prompting the laboratory to extend culture times) and a complete medical history inquiry are instrumental in the early diagnosis of *M*. marinum infections.

Future research should focus on the epidemiology, pathogenic mechanisms, and treatment strategies for *M*. marinum infections. This includes studies on antibiotic resistance and improving diagnostic methods for earlier identification and treatment of infections. Additionally, research should explore better ways to educate the public and medical professionals to enhance awareness and prevention of *M*. marinum infections.

## Data availability statement

The datasets presented in this study can be found in online repositories. The names of the repository/repositories and accession number(s) can be found in the article/supplementary material.

## Ethics statement

Written informed consent was obtained from the individual(s) for the publication of any potentially identifiable images or data included in this article.

## Author contributions

CL: Conceptualization, Writing – review & editing, Resources. JH: Conceptualization, Writing – original draft. MS: Investigation, Writing – review & editing. JY: Investigation, Writing – review & editing. CZ: Writing – review & editing, Resources. YH: Writing – review & editing. ZF: Writing – review & editing. RJ: Writing – review & editing. YS: Writing – review & editing. WG: Writing – review & editing. HZ: Writing – review & editing. ZZ: Writing – review & editing. LZ: Writing – review & editing.
